# Decreased plasma fetuin-A level as a novel bioindicator of poor prognosis in community-acquired pneumonia: A multi-center cohort study

**DOI:** 10.3389/fmed.2022.807536

**Published:** 2022-07-29

**Authors:** Lili Zhao, Ying Shang, Qiongzhen Luo, Xinqian Ma, Wentao Ni, Yukun He, Donghong Yang, Yu Xu, Zhancheng Gao

**Affiliations:** ^1^Department of Respiratory and Critical Care Medicine, Peking University People's Hospital, Beijing, China; ^2^Department of Respiratory and Critical Care Medicine, School of Clinical Medicine, Beijing Tsinghua Changgung Hospital, Tsinghua University, Beijing, China; ^3^Department of Respiratory and Critical Care Medicine, Beijing Jishuitan Hospital, Beijing, China

**Keywords:** community-acquired pneumonia, fetuin-A, prognosis, mortality, diagnosis

## Abstract

**Background:**

Community-acquired pneumonia (CAP) is a respiratory disease that frequently requires hospital admission, and is a significant cause of death worldwide. Plasma fetuin-A levels were significantly lower in patients with sepsis, but data regarding CAP are scarce. This study aimed to evaluate the usefulness of fetuin-A as a prognostic biomarker of CAP.

**Methods:**

A multicenter cohort study on CAP was conducted between January 2017 and December 2018. Demographic and clinical data were recorded for all enrolled patients. Plasma fetuin-A levels were determined using a quantitative enzyme-linked immunosorbent assay. A Cox proportional hazards regression analysis was used to analyse the effect of variables on 30-day mortality. A logistic regression analysis was performed to assess risk factors associated with severe CAP (SCAP) and 30-day mortality. A receiver operating characteristic (ROC) curve was used to verify the association between variables and CAP prognosis. Correlations were assessed using Spearman's test. Survival curves were constructed and compared using the log-rank test.

**Results:**

A total of 283 patients with CAP were enrolled in this study. Fetuin-A levels were decreased in patients with CAP, especially in SCAP and non-survivors. A cox regression analysis showed that CURB-65 and fetuin-A levels were independent prognostic indicators of 30-day mortality. *Via* a multiple logistic regression analysis, plasma level of fetuin-A (<202.86 mg/L) was determined to be the strongest independent predictor of 30-day mortality considered (odds ratio, 57.365), and also was also determined to be an independent predictor of SCAP. The area under the curve (AUC) of fetuin-A for predicting 30-day mortality was 0.871, and accuracy was high (*P* < 0.05). Plasma fetuin-A levels were negatively correlated with WBC, NE%, Glu, CRP, PCT, CURB-65, and pneumonia severity index scores and positively correlated with albumin level. Kaplan–Meier curves showed that lower plasma levels of fetuin-A levels were associated with increased 30-day mortality levels (*P* < 0.0001).

**Conclusion:**

Plasma fetuin-A levels were decreased in patients with CAP. Fetuin-A can reliably predict mortality in patients with CAP, and is a useful diagnostic indicator of SCAP.

## Introduction

Community-acquired pneumonia (CAP) is a common respiratory infectious disease that frequently requires hospitalization. The economic burden of CAP, and its morbidity and mortality levels are high, with ~10% of hospitalized patients requiring admission to an intensive care unit (ICU) (1, 2). Hospital mortality among patients with SCAP as high as 50% (2, 3). Early diagnosis and risk stratification for CAP reduces SCAP incidence and mortality but is particularly challenging for physicians (4, 5).

PSI and CURB-65 (confusion, urea, respiratory rate, blood pressure, age 65 and older) are the most widely used risk stratification tests (6, 7). PSI stratifies individuals with CAP into five groups based on short-term mortality risk. The determination of a patient's PSI score requires knowledge of many indices, and score calculation takes a long time, therefore its clinical applicability is limited. CURB65 is easier to determine than PSI but predicts mortality less sensitively. Furthermore, neither score assesses the host inflammatory response, which is may helpful for evaluating CAP prognosis.

Currently, traditional and novel predictors including white blood cell (WBC) count (8), C-reactive protein (CRP) (9), procalcitonin (PCT) (10), soluble triggering receptors expressed on myeloid cells-1 (11), pro-adrenomedullin (12), and progranulin (13) levels have been shown to be valuable prognostic indicators in patients with CAP. However, their sensitivity and specificity for prognosis prediction are variable and insufficient, therefore, the identification of new predictors to improve risk stratification in patients with CAP is needed (14).

Fetuin-A, also known as a 2-Heremans-Schmid glycoprotein, was first identified in fetal serum. It is mainly synthesized by liver parenchymal cells and plays important roles in multiple biological functions, including calcification, cardiovascular diseases, insulin sensitivity, apoptosis, tumor development, diabetes, obesity, and fatty liver disease (15, 16). Plasma fetuin-A levels are reduced in patients with sepsis, suggesting that levels of fetuin-A in plasma may serve as a useful indicator of CAP prognosis (17, 18). Few prior studies have assessed plasma fetuin-A levels in patients with CAP (19, 20).

Based on previous studies, we hypothesized that plasma fetuin-A levels might be correlated with CAP risk and prognosis. Thus, the aim of this study was to clarify the precise role of fetuin-A in CAP. To determine the effectiveness of fetuin-A as an indicator of the risk of death in CAP and CAP severity, 30-day survival was the primary outcome, and SCAP was the secondary outcome assessed.

## Materials and methods

### Study design

#### Participating patients and controls

This was an observational prospective cohort study that assessed patients with CAP from January 2017 to September 2018 who were hospitalized at Peking University People's Hospital, the Second Hospital of Jilin University, West China Hospital, Shanghai Pulmonary Hospital, Tibet Autonomous Region People's Hospital, and Fujian Provincial Hospital (ClinicalTrials.gov ID, NCT03093220). This study was approved by the Medical Ethics Committee of Peking University People's Hospital (approval no.: 2016PHB202-01). Our healthy controls were volunteers from staff of Peking University People's Hospital. Informed consent was obtained from all participating patients. The trial was funded by the Chinese Science and Technology Key Project (2017ZX10103004-006) and the National Key Research and Development Programme of China (2016YFC0903800). The funder had no role in the study design, data analysis, or outcome assessment.

#### Definition of CAP and SCAP

The study included subjects older than 18 years meeting the following diagnostic criteria specified in diagnosis of community-acquired pneumonia in adults (21): (1) onset of symptoms in the community; (2) a chest radiograph showing either a new patchy infiltrate, leaf or segment consolidation, ground glass opacity, or interstitial change; (3) either (a) the presence of cough, sputum production, and dyspnea; (b) core body temperature > 38.0°C; (c) auscultatory findings of abnormal breath sounds and rates; or (d) peripheral white blood cell count >10 × 10^9^/L or < 4 × 10^9^/L. The following exclusion criteria were applied: (1) age <18 years; (2) patients with active tuberculosis; (3) pregnancy; (4) malignant lung tumor; (5) pulmonary interstitial disease; (6) pulmonary embolism; (7) pulmonary vasculitis; and (8) human immunodeficiency virus infection. Severe CAP (SCAP) was diagnosed based on the presence of at least one major criterion or at least three minor criteria. The criteria are almost identical to those for diagnosis CAP & SCAP by the American Thoracic Society (22).

#### Study aims

In the study, 30-day survival was the primary outcome assessed, and SCAP was the secondary outcome considered.

### Data collection and follow-up

Clinical characteristics including sex, age, and the presence of comorbidities (chronic cardiac disease, chronic renal disease, chronic liver disease, high blood pressure, diabetes mellitus, and malignant diseases), and laboratory findings including WBC count, percentage of neutrophils (NE%), blood biochemistry, blood gas analysis, CRP, and PCT were recorded within 24 h of admission. Simultaneously, PSI and CURB-65 scores were calculated for all patients. Drug treatment after admission, complications, ventilation, survival time, mortality and SCAP were also recorded. The loop-mediated isothermal amplification method was used to detect bacteria, viruses, and fungi from respiratory-derived specimens in patient.

### Measurement of fetuin-A levels

Blood samples were collected in anticoagulant tubes within 24 h of admission, and centrifuged immediately. Supernatants of plasma samples were stored at −80°C for further analysis. Plasma fetuin-A levels were measured using quantitative ELISA kits (DFTA00, R&D Systems, Minneapolis, MN, USA) in accordance with the manufacturer's instructions. Briefly, the absorbance of standard and plasma samples at 450 nm was measured using a Multiskan FC system (Thermo, Waltham, MA, USA) with the correction wavelength set at 570 nm. A standard curve was created using a standard sample using a four-parameter logistic curve fit, and plasma fetuin-A levels were calculated based on the curve created.

### Statistical analysis

Continuous variables are presented as mean ± standard deviation or median (interquartile range), and were assessed using an unpaired *t*-test or Mann–Whitney *U*-test, as appropriate. Categorical variables are presented as frequencies and percentages, and were analyzed using either chi-square, Fisher's exact, or correction for continuity chi-square tests, as appropriate. Cox proportional hazards regression analyses were used to analyse effects of variables on 30-day survival.

Fetuin-A levels were categorized into four groups according to percentiles, as follows: group 1, ≥ 365.92 mg/L (50th percentile); group 2, 271.54–365.92 mg/L (25th percentile); group 3, 202.86–271.54 mg/L (10th percentile); group 4, <202.86 mg/L (5th percentile). Using group 1 as a reference, logistic regression models were used to calculate odds ratios (ORs) for groups 2, 3, and 4. Further, ORs for groups IV and V of PSI were calculated using Groups I-III (PSI) as reference, and OR for scores (2–5) of CURB-65 were calculated using 0-1 (CURB-65) as reference. Logistic regression analyses were used to assess effects of an array of variables on SCAP. Receiver operating characteristic (ROC) curves were used to evaluate the accuracy by which different variables predicted 30-day mortality in patients with CAP. Correlations between non-normally distributed variables were assessed using the Spearman's rho test. Kaplan–Meier methods were used to build 30-day survival curves, and values were compared using the log-rank test.

All primary and secondary outcome analyses were performed using SPSS software (version 20.0; SPSS Inc., Chicago, IL, USA), GraphPad Prism version 8 software (GraphPad Software, La Jolla, CA, USA), and MedCalc statistical software v.15.2.2 (MedCalc). A two-sided value of *P* < 0.05 was considered statistically significant (^*^P < 0.05, ^**^P < 0.01, ^***^P < 0.001, ^****^P < 0.0001).

The sample size and power calculation for the Cox proportional hazards regression of the survival analysis was as follows:


$$N=\frac{{\left(Z_{1-\text{α}/2}+{\text{Z}}_{1\text{-β}}\right)}^{2}}{\text{P}\left({\text{1-R}}^{\text{2}}\right)\sigma^{\text{2}}{\text{B}}^{2}}$$

In this study, we set the two-sided significance level to α = 0.05. According to the above formula, with a sample size of 283, the standard deviation (Z1-α/2) was 1.96, 30-day mortality *P* was 0.0671, the R-squared value of fetuin-A with CURB-65 was 0.094, the standard deviation of fetuin-A σ was 137.55, the log hazard ratio of fetuin-A (B) was −0.012, and the calculated power was 0.999.

## Results

Demographic characteristics and laboratory findings of enrolled patients with CAP and healthy controls are presented in Table 1. There were 85 missing values in overall 9,339 statistical values, with a missing rate of 0.91%. Using the MCAR test, the missing values were shown to be completely missing at random (*P* = 0.381). Important indicators (including survival time, 30-day survival outcomes, SCAP, plasma fetuin-A levels, PSI and CURB65 scores) were free of missing values in our study. Therefore, the missing values were directly deleted in follow-up analysis. The 283 patients enrolled from January 2017 to September 2018 were divided into survivor and non-survivor groups. Thirty-day mortality in patients with CAP was 6.71% (*n* = 19). There were no significant between-group differences observed in terms of sex, age, comorbidities, WBC count, hemoglobin level, platelet count, glucose level, or antibiotic use. Laboratory analyses revealed that patients in the non-surviving group had higher NE%, blood urea nitrogen, CRP, and PCT, and lower levels of albumin (ALB) than survivors of CAP. No statistically significant between-group differences regarding bacterial, virus, or fungal detection rates were detected using the pathogen culture combined Loop-mediated isothermal amplification (LAMP) method. Non-survivors more frequently received antiviral drugs, corticosteroids, and ventilation treatments during hospitalization than survivors (*P* < 0.0001, *P* = 0.0001, *P* < 0.0001, respectively), and were more likely to develop complications and SCAP (*P* < 0.0001) and admitted to the ICU (*P* < 0.0001). PSI and CURB-65 scores throughout the 24 h following hospital admission of the non-survivor group were significantly higher than those of the survivor group (*P* < 0.0001 for both; Table 1).

**Table 1:** Clinical characteristics and laboratory findings of survivors and non-survivors.

	**Survivors (I)**	**Non-survivors (II)**	**Control**	***P*** **-value**
	***n*** = **264 (93.29%)**	***n*** = **19 (6.71%)**	***n*** = **36**	**(I vs. II)**
Male sex (%)	161 (60.98%)	13 (68.42%)	17 (47.22%)	0.520
Age (years)	66 (54–76)	70(65–78)	47(42–52)	0.081
**Comorbidities, *n* (%)**				
Heart disfunction	23 (8.71%)	0 (0.00%)	2(5.56%)	0.381
Chronic renal disease	15 (5.68%)	0 (0.00%)	1 (2.78%)	0.609
Liver disease	12(4.55%)	0 (0.00%)	1(2.78%)	1.000
Diabetes mellitus	59 (22.35%)	2 (10.53%)	2 (5.56%)	0.357
High pressure	91 (34.47%)	5 (26.32%)	5 (13.89%)	0.468
**Laboratory findings**				
WBC count (×10^3^/mm^3^)	6.80 (5.00–10.60)	10.70(4.40–17.20)	5.42 (4.76–7.31)	0.199
NE%	73.90 (61.20–82.80)	87.10 (83.60–93.00)	53.05 (50.40–58.03)	<0.0001
Hemoglobin level (g/dL)	128.00 (125.00–148.00)	132.00 (112.00–148.00)	148.00 (130.50–154.75)	0.323
Platelet count (×10^3^/mm^3^)	203.00 (147.00–273.00)	186.00 (88.00–311.00)	239.00 (224.00–273.25)	0.918
Glucose (mmol/L)	5.60 (4.84–7.03)	7.00 (4.85–8.64)	5.36 (4.87–5.90)	0.107
Albumin (g/L)	36.00 (32.00–39.00)	27.80 (23.40–33.00)	45.40 (43.23–46.78)	0.0001
Blood urea nitrogen (mmol/L)	4.90 (3.78–6.40)	5.90 (5.00–10.70)	NA	0.007
CRP (mg/L)	39.70 (8.50–114.75)	136.00 (88.62–185.00)	NA	0.0004
PCT (μg/L)	0.11 (0.05–0.63)	1.30 (0.18–4.16)	NA	0.001
**Complications, *n* (%)**				
Sepsis	22 (8.33%)	12 (63.16%)	NA	<0.0001
Pleural effusion	91 (34.47%)	11 (57.89)	NA	0.040
ARDS	5 (1.89%)	11(57.89%)	NA	<0.0001
Confusion	8 (3.03%)	4 (21.05%)	NA	0.002
Non-invasive ventilation, *n* (%)	146 (55.30%)	2 (10.53%)	NA	0.0002
Invasive ventilation, *n* (%)	15 (5.68%)	17 (89.47%)	NA	<0.0001
ICU admission, *n* (%)	35 (13.25%)	16 (84.21%)	NA	<0.0001
**Pathogens *n* (%)**				
Bacteria	60 (22.73%)	3 (15.79%)	NA	0.677
Virus	75 (28.41%)	4 (21.05%)	NA	0.490
Fungus	19 (7.20%)	1 (5.26%)	NA	1.000
Mixed	82 (31.06%)	8 (42.11%)	NA	0.318
**Drug treatment, *n* (%)**				
Antibiotics	259 (98.11%)	19 (100.00%)	NA	1.000
Antiviral drugs	31 (11.74%)	13 (68.42%)	NA	<0.0001
Corticosteroids	38 (14.39%)	9 (47.37%)	NA	0.001
PSI	78 (59–96)	96 (85–118)	NA	0.0004
CURB-65	1 (0–1)	2 (1–2)	NA	<0.0001
Severe CAP, *n* (%)	39 (14.77%)	18 (94.74%)	NA	<0.0001

Data are presented as means ± standard deviation from the mean, or median (interquartile range) or n (%). WBC, White blood cell; NE%, percentage of neutrophils; CRP, C-reactive protein; PCT, procalcitonin; ARDS, Acute Respiratory Distress Syndrome; PSI, Pneumonia Severity Index; CURB-65 confusion, urea >7 mmol/L, respiratory rate ≥ 30 breaths/min, low blood pressure, and age ≥ 65 years; NA, not applicable.

### A comparison of plasma fetuin-A levels

**Figure 1:**
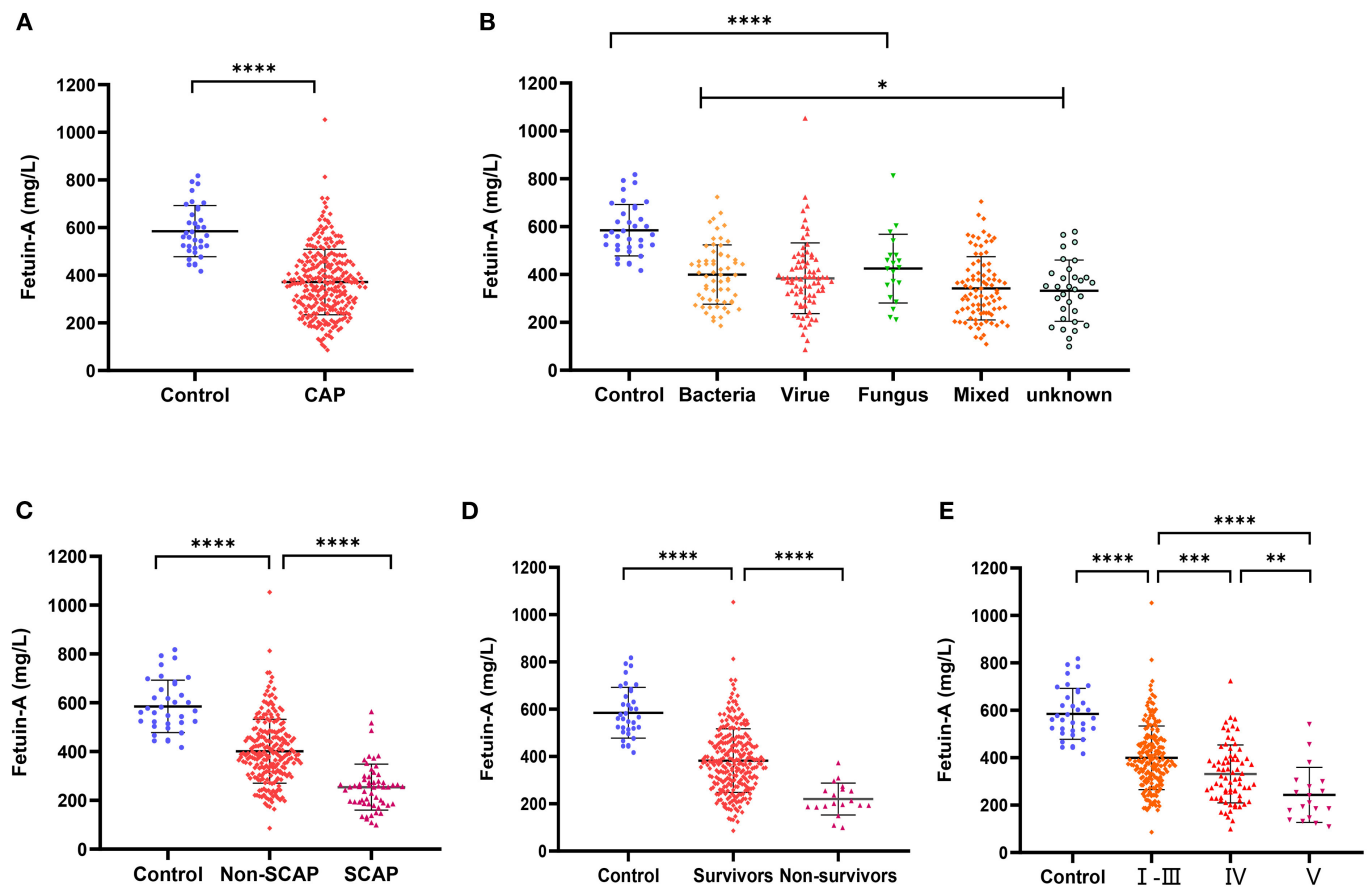
Levels of plasma Fetuin-A in groups of patients considered. **(A)** Levels of Fetuin-A in patients with community-acquired pneumonia (CAP) and healthy individuals. **(B)** Comparison of Fetuin-A levels in CAP patients with different disease etiologies. **(C)** Levels of Fetuin-A in patients with and without severe community-acquired pneumonia (SCAP). **(D)** Levels of Fetuin-A among CAP survivors and non-survivors. **(E)** Levels of Fetuin-A in patients classified as high (V), middle (IV), and low risk based on PSI (*P < 0.05, **P < 0.01, ***P < 0.001, ****P < 0.0001).

As shown in Figure 1A, plasma fetuin-A levels in patients with CAP were 371.8 ± 137.6 mg/L, a value significantly lower than that of healthy individuals (585.1 ± 107.2 mg/L, *P* < 0.0001). Pathogens detected in patients with CAP were classified as follows: bacterial, viral, fungal, mixed, or of unknown type. Levels of fetuin-A in patients with different causative pathogens of CAP differed using ANOVA anlysis method (*P* < 0.05). Fetuin-A levels were significantly higher in healthy individuals than patients with CAP caused by each pathogen considered (*P* < 0.0001 for each comparison; Figure 1B).

Various metrics were applied to assess CAP severity. Patients with SCAP had lower levels of Fetuin-A (254.1 ± 94.35 mg/L) than both patients with CAP who did not have SCAP (401.4 ± 130.8 mg/L) and those of the control group (585.1 ± 107.2 mg/L; *P* < 0.001 for both). Levels of fetuin-A in control, non-SCAP, and SCAP groups tended to decrease (*P* < 0.001; Figure 1C). Similarly, the mean fetuin-A level of the non-survivor group was 219.9 ± 67.56 mg/L, notably lower than that of the survivor group (382.7 ± 134.9 mg/L; *P* < 0. 0001; Figure 1D). Plasma fetuin-A levels were significantly lower in patients classified *via* PSI as high-risk (V) than in those classified as middle (IV) (*P* < 0.01) and low risk (I–III) (*P* < 0.0001; Figure 1E). Similarly, fetuin-A levels in middle-risk PSI (IV) patients were significantly lower than those who were determined to be low risk (I–III) (*P* < 0.001).

### Prognostic power of plasma fetuin-A level for assessing 30-day mortality risk in patients with CAP

Independent predictors were subjected to univariate Cox proportional hazard regression analyses to investigate their possible associations with 30-day mortality. Results revealed that WBC, NE%, ALB, CRP, PSI, CURB-65, and fetuin-A were significantly associated with high-risk ratios. When these significant independent variables were integrated into a multivariate Cox proportional hazards regression analysis, only CURB-65 [hazard ratio (HR): 1.598, 95% CI: 1.029–2.483] and fetuin-A (HR: 0.989, 95% CI: 0.984–0.994) were independent predictors of 30-day mortality (*P* = 0.037 and *P* < 0.0001, respectively; Table 2).

**Table 2:** Cox regression analysis of risk factors associated with 30-day mortality.

	**Univariate analysis**		**Multivariate analysis**	
	**Hazard ratio (95% CI)**	***P*** **-value**	**Hazard ratio (95% CI)**	***P*** **-value**
Male sex	1.375 (0.522–3.617)	0.519		
Age	1.030 (0.998–1.063)	0.071		
WBC	1.064 (1.011–1.120)	0.017		
NE%	1.096 (1.043–1.150)	0.0002		
Hemoglobin level	1.004 (0.986–1.023)	0.663		
Platelet count	1.001 (0.996–1.005)	0.821		
Glucose	1.068 (0.943–1.209)	0.301		
Albumin	0.870 (0.809–0.936)	0.0002		
Blood urea nitrogen	1.003 (0.987–1.019)	0.718		
CRP	1.006 (1.003–1.009)	<0.0001		
PCT	0.997 (0.936–1.063)	0.934		
PSI	1.023 (1.010–1.036)	0.0003		
CURB-65	2.490 (1.601–3.873)	<0.0001	1.599 (1.029–2.483)	0.037
Fetuin-A	0.987 (0.982–0.992)	<0.0001	0.989 (0.984–0.994)	<0.0001

CI, Confidence interval; WBC, White blood cell; NE%, percentage of neutrophils; CRP, C-reactive protein; PCT, procalcitonin; PSI, Pneumonia Severity Index; CURB-65, confusion, urea >7 mmol/L, respiratory rate ≥ 30 breaths/min, low blood pressure and age ≥ 65 years.

Logistic regression analyses of different stratifications of PSI, CURB-65, and fetuin-A concentrations were associated with non-survivors (Table 3). As the concentration of fetuin-A decreased, mortality increased, and the OR for group 3 was 19.189 (*P* = 0.008) and for group 4 was 78.889 (*P* < 0.0001) when compared with group 1. Fetuin-A functioned as an indicator of mortality in a manner similar to PSI and CURB-65. After adjusting for sex, age, WBC, NE%, hemoglobin level, platelet count, glucose, ALB, blood urea nitrogen, CRP, and PCT, ORs (95% CI) calculated for group 3 and group 4 were 10.609 (1.071–105.052) and 57.365 (5.768–570.488), respectively, which showed that fetuin-A concentrations were significantly elevated versus group 1 (*p* = 0.044 and *p* = 0.001, respectively).

**Table 3:** Logistic regression analysis of risk factors associated with non-survivors.

	**Univariate analysis**		**Multivariate analysis** ^#^	
	**Odd ratio (95% CI)**	***P*** **-value**	**Odd ratio (95% CI)**	***P*** **-value**
**PSI**				
PSI (I–III)	Reference			
PSI (IV)	3.304 (1.152–9.473)	0.026		
PSI (V)	7.048 (1.852–26.819)	0.004		
**CURB-65**				
CURB-65 (0–1)	Reference			
CURB-65 (2–5)	6.875 (2.615–18.073)	<0.0001	4.716 (1.476–15.064)	0.009
**Fetuin-A**				
Group 1 (≥365.92)	Reference			
Group 2 (≥271.54, <365.92)	6.358 (0.649–62.270)	0.112	4.464 (0.432–46.124)	0.209
Group 3 (≥202.86, <271.54)	19.189 (2.175–169.295)	0.008	10.609 (1.071–105.052)	0.044
Group 4 (<202.86)	78.889 (9.533–652.851)	<0.0001	57.365 (5.768–570.488)	0.001

CI, Confidence interval; WBC, White blood cell; NE%, percentage of neutrophils; CRP, C-reactive protein; PCT, procalcitonin; PSI, Pneumonia Severity Index; CURB-65, confusion, urea >7 mmol/L, respiratory rate ≥30 breaths/min, low blood pressure and age ≥ 65 years. ^#^Adjusted for Male sex, Age, WBC, NE%, Hemoglobin level, Platelet count, Glucose, Albumin, Blood urea nitrogen, CRP, PCT.

**Figure 2:**
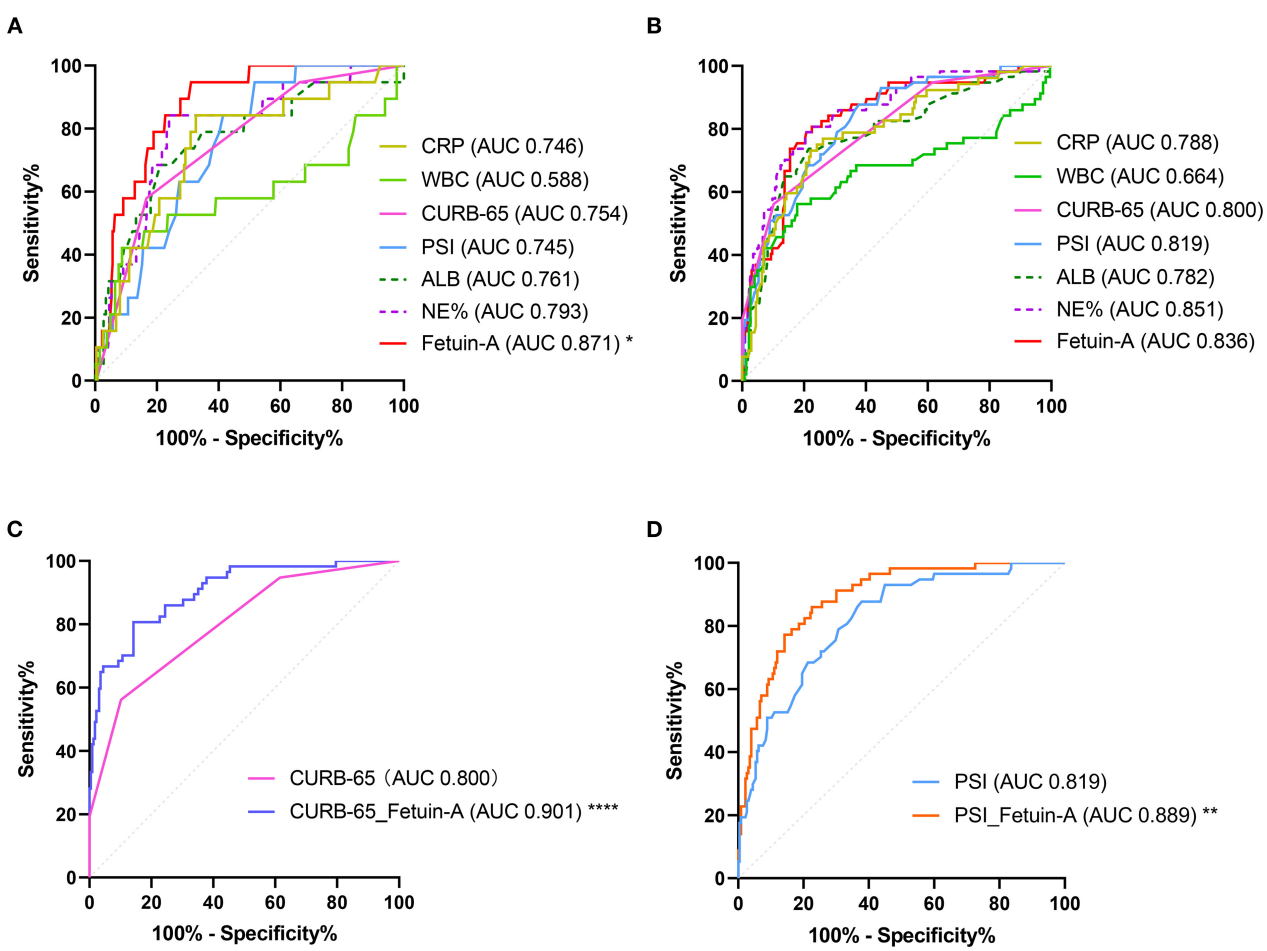
Receiver operating characteristic (ROC) analysis of fetuin-A level for predicting **(A)** 30-day mortality and **(B)** SCAP. In **(C)**, a comparison of use of a combination of Fetuin-A and CURB-65 vs. CURB-65 alone is shown. In **(D)**, a comparison of the use of a combination of Fetuin-A and PSI use vs. PSI use alone is shown (*P < 0.05, **P < 0.01, ****P < 0.0001).

ROC showed that plasma Fetuin-A levels had the highest accuracy for predicting the 30-day mortality in patients with CAP among all indicators assessed, with an AUC of 0.871 (95% CI: 0.826–0.907). For fetuin-A, the optimal cut-off value was determined to be 309 mg/L, which was significantly higher that of other predictors of 30-day mortality (*P* < 0.05; Figure 2A, Supplementary Table 1).

### Association between plasma fetuin-A level and CAP severity

Clinical characteristics of enrolled patients with and without SCAP are presented in Supplementary Table 2. Results showed that WBC, NE%, glucose, ALB, CRP, PCT, PSI, CURB-65, and fetuin-A were risk factors associated with SCAP *via* univariate logistic analyses. After adjusting for the nine predictors, three were included in the multivariate logistic analysis using the forward conditional method. ORs of NE%, CURB-65, and fetuin-A were 1.091, 7.745, and 0.990, respectively (<0.0001 for all; Table 4). Logistic regression analysis for different stratifications of fetuin-A concentrations associated with SCAP (Supplementary Table 3) revealed that as the concentration of fetuin-A decreased, the likelihood of occurrence of SCAP increased. ROC analysis showed that plasma fetuin-A level had an AUC of 0.836 for predicting 30-day mortality in CAP patients. However, no significant difference was observed when it was compared to CURB-65 and PSI (Figure 2B, Supplementary Table 4). Combining fetuin-A with CURB-65 and PSI significantly improved predictive accuracy (*p* < 0.0001 and *p* < 0.01, respectively; Figures 2C,D, Supplementary Table 4).

**Table 4:** Logistic regression analysis of risk factors associated with SCAP.

	**Univariate analysis**		**Multivariate analysis**	
	**Odd ratio (95% CI)**	***P*** **-value**	**Odd ratio (95% CI)**	***P*** **-value**
Male sex	2.227 (1.153–4.303)	0.017		
Age	1.007 (0.989–1.025)	0.471		
WBC	1.120 (1.064–1.180)	<0.0001		
NE%	1.137 (1.094–1.182)	<0.0001	1.091 (1.045–1.139)	<0.0001
Hemoglobin level	0.997 (0.985–1.009)	0.620		
Platelet count	0.998 (0.995–1.001)	0.258		
Glucose	1.113 (1.014–1.220)	0.024		
Albumin	0.836 (0.789–0.885)	<0.0001		
Blood urea nitrogen	1.006 (0.993–1.019)	0.399		
CRP	1.012 (1.008–1.016)	<0.0001		
PCT	1.103 (1.047–1.163)	0.0002		
PSI	1.045 (1.031–1.059)	<0.0001		
CURB-65	6.101 (3.658–10.176)	<0.0001	7.745 (3.628–16.533)	<0.0001
Fetuin-A	0.987 (0.983–0.991)	<0.0001	0.990 (0.985–0.995)	<0.0001

CI, Confidence interval; WBC, White blood cell; NE%, percentage of neutrophils; CRP, C-reactive protein; PCT, procalcitonin; PSI, Pneumonia Severity Index; CURB-65, confusion, urea >7 mmol/L, respiratory rate ≥30 breaths/min, low blood pressure and age ≥ 65 years.

### Associations between predictors levels

**Figure 3:**
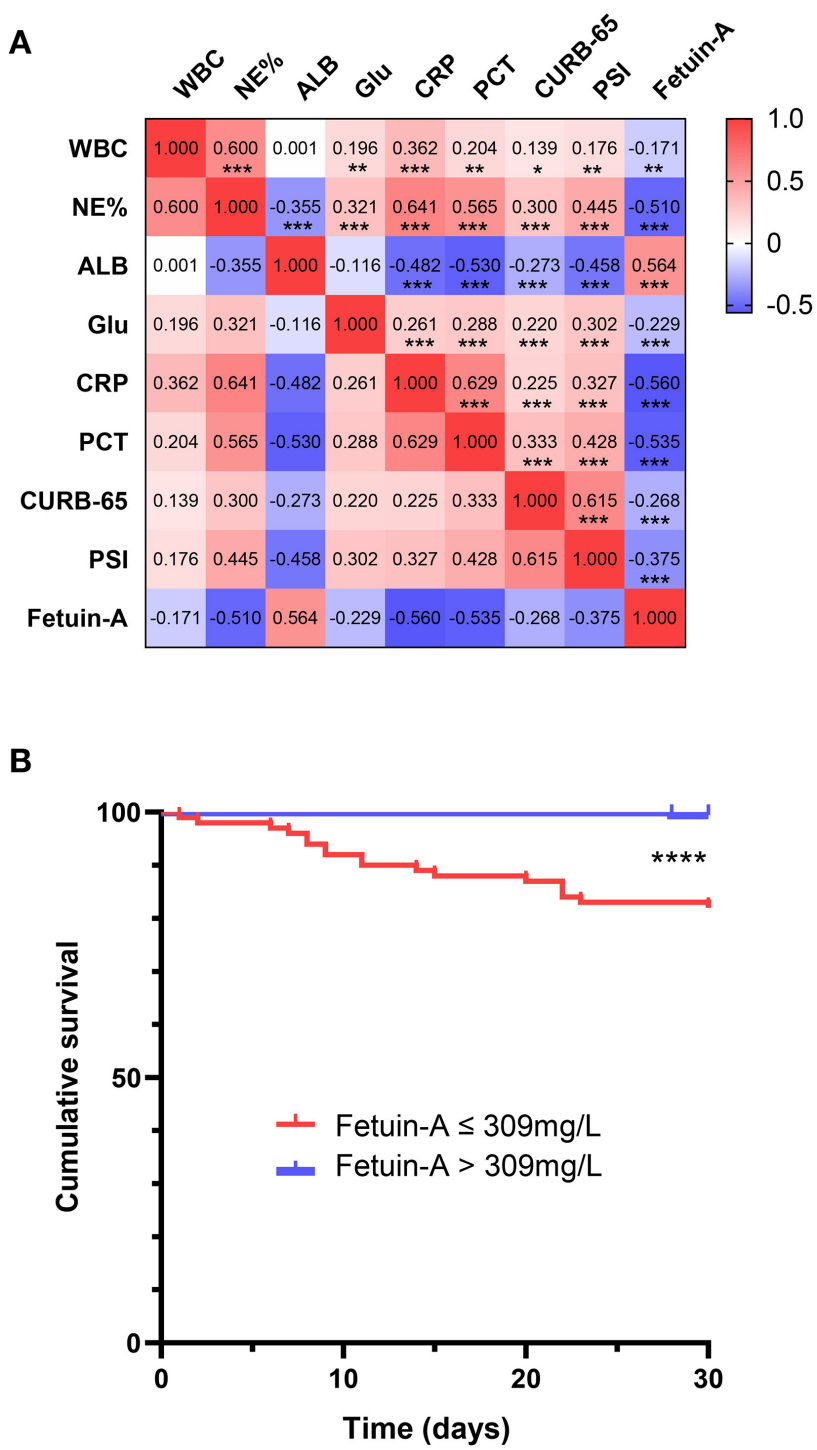
Correlation and survival analysis **(A)** of fetuin-A level vs. multiple clinical predictors of community-acquired pneumonia (CAP). **(B)** A Kaplan–Meier analysis of 30-day mortality in CAP patients is shown *P < 0.05, **P < 0.01, ***P < 0.001, ****P < 0.0001.

Relationships between plasma fetuin-A levels and clinical parameters were investigated using Spearman's correlation analyses. The resultant correlation matrix is shown in Figure 3A. Fetuin-A was found to be positively correlated with ALB, and negatively correlated with WBC (*P* < 0.01), NE%, Glu, CRP, PCT, CURB-65, and PSI score (*P* < 0.001 for all except WBC).

### Kaplan–Meier survival analysis of Fetuin-A in CAP patients

Kaplan–Meier curves were used to assess whether plasma fetuin-A level predicted 30-day mortality in patients with CAP (Figure 3B). Patients were divided into two groups based on ROC curves thresholds, as follows: fetuin-A ≤ 309 mg/L and fetuin-A > 309 mg/L. Risk of death in patients with fetuin-A levels ≤ 309 mg/L was significantly higher than that of patients with fetuin-A levels > 309 mg/L (*P* < 0.0001).

### Comparison of plasma fetuin-A levels at discharge and admission

**Figure 4:**
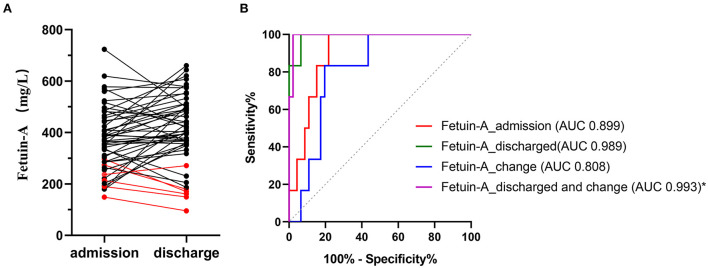
Levels of paired plasma Fetuin-A in 52 patients with CAP at admission and discharge **(A)**. Red represents dead patients and black represents recovered patients. **(B)** ROC analysis of different fetuin-A level for predicting 30-day mortality. Fetuin-A _ admission represents fetuin-A levels at admission; fetuin-A _ discharge represents fetuin-A levels at discharge; fetuin-A _ change represents plasma fetuin-A level at discharge minus that of admission; fetuin-A _ Discharge and change represents combination of fetuin-A at discharge and fetuin-A _ change.

We only collected plasma from a small subset of patients with CAP in the 24 h before discharge (including recovery and death). Fetuin-A levels were detected in the matched plasma of these 52 patients. As shown in Figure 4A, red represents dead patients and black represents recovered patients. A total of 6 patients died, of which 5 patients had decreased plasma fetuin-A levels and fetuin-A levels were <200 mg/L before death. Plasma fetuin-A level of another dead patient increased slightly and was still at a low level of 271 mg/L. The plasma fetuin-A levels increased or were stable in most of recovered patients, while 3 recovered patients had lower fetuin-A levels of <300 mg/L. The mean plasma fetuin-A level in recovered patients was 442 mg/L, which was higher than that of 390 mg/L in patients with CAP at admission (*P* = 0.03), but was still lower than that of 585 mg/L in healthy people (*P* < 0.0001).

### ROC of plasma fetuin-A level for assessing 30-day mortality risk in 52 patients with CAP

We re-performed the analysis of plasma fetuin-A levels at admission and discharge in 52 patients to assess 30-day mortality. ROC showed that AUC of plasma fetuin-A levels at admission and discharge for predicting the 30-day mortality were 0.899 and 0.989, respectively. Fetuin-A _ change representes plasma fetuin-A level at discharge minus that of admission. ROC showed that combining fetuin-A at discharge with fetuin-A _ change (fetuin-A _ discharge and change) had the highest accuracy for predicting the 30-day mortality in patients with CAP, with an AUC of 0.993 (95% CI: 0.932–1.000), and it significantly improved predictive accuracy compared to fetuin-A _ admission (*p* = 0.028; Figure 4B, Supplementary Table 5).

## Discussion

In this prospective study of 283 patients with CAP, 30-day mortality was 6.71%, and 20.14% of patients developed SCAP. The following eight major findings were revealed: (1) plasma fetuin-A levels were reduced in patients with CAP vs. healthy individuals, especially in non-survivors and those with SCAP; (2) plasma fetuin-A level was an independent predictor of 30-day mortality; (3) the OR of the high fetuin-A level group was significantly increased vs. the low fetuin-A concentration group for predicting 30-day mortality; (4) reductions in fetuin-A levels were significantly associated with high-risk ratios of SCAP when values were adjusted for WBC, NE%, Glu, ALB, CRP, PCT, PSI, and CURB-65, and as the concentration of fetuin-A decreased, the likelihood SCAP development increased; (5) plasma fetuin-A levels most accurately predicted 30-day mortality when compared with all parameters assessed, and the accuracy increase was statistically significant; (6) combining fetuin-A with clinical severity score significantly improved SCAP predictive accuracy; (7) plasma fetuin-A levels were positively correlated with ALB and negatively correlated with WBC, NE%, Glu, CRP, PCT, CURB-65, and PSI scores; and (8) mortality rate was significantly higher in patients with fetuin-A levels <309 mg/L than in those with fetuin levels ≥309 mg/L. Simultaneously, comparison of plasma fetuin-A levels at discharge and admission of 52 patients with CAP, plasma fetuin-A continued to decrease during the progression of CAP and <200 mg/L, patients were at extremely high risk of death. Taken together, study findings suggest that plasma fetuin-A level is an effective predictor of 30-day mortality and disease severity in patients with CAP.

An ANOVA analysis of plasma fetuin-A levels in patients infected with different causative agents of CAP revealed that pathogen type affected fetuin-A level (Figure 1B). However, no significant differences in fetuin-A levels were observed when viral and mixed infection groups were compared. Therefore, plasma fetuin-A levels were not determined to be clinically useful for evaluating pathogen type.

Fetuin-A has been widely studied in a variety of diseases including non-alcoholic fatty liver disease (23), diabetes (24), cardiovascular disease (25), sepsis (17, 18), periodontitis (26, 27), osteoarthritis (28), chronic kidney disease (29), inflammatory bowel disease (30), and encapsulating peritoneal sclerosis (31). Many of these diseases are associated with inflammation. Serum fetuin-A levels are significantly reduced in patients with sepsis and septic shock, and non-survivors have lower fetuin-A levels than survivors. Fetuin-A was negatively correlated with CRP and procalcitonin, but positively correlated with albumin. A Kaplan-Meier estimate of mortality in 102 septic patients showed that subjects whose serum fetuin-A level recovered rapidly between baseline and day 7 had improved rates of survival than those whose fetuin-A level did not rapidly recover (32). However, another study of sepsis in preterm neonates showed that fetuin-A level is not a useful prognostic indicator (18). Perhaps the differing conclusions drawn by the two studies were related to the different research subjects evaluated; adults and newborns.

Salivary and serum fetuin-A levels were significantly lower in patients with stages II–III periodontitis than healthy subjects, and fetuin-A levels diminished as the severity of periodontal inflammation increased (27). Salivary fetuin-A levels were significantly lower in patients with chronic periodontitis than healthy individuals, and a negative correlation between fetuin-A and CRP levels was observed (26). In patients with Crohn's disease and ulcerative colitis, serum fetuin-A levels were significantly lower than that of healthy controls. Patients with inflammatory bowel disease could be distinguished from HCs with approximately 90% sensitivity and specificity *via* ROC analysis (30).

Few studies have examined the expression of fetuin-A in pneumonia. Only one study in children showed that serum fetuin-A levels in invasive pneumococcal disease with hemolytic uremic syndrome were significantly lower than in patients with lobar pneumonia and healthy controls; however, fetuin-A levels in lobar pneumonia did not significantly differ from those in healthy controls (19). To date, no studies have examined fetuin-A levels in adult patients with pneumonia. Our study confirmed that plasma fetuin-A levels were significantly decreased in patients with CAP. Cox proportional regression analysis showed that fetuin-A was an independent predictor of 30-day mortality in hospitalized patients with CAP after justing for other factors. Fetuin-A level can be used to successfully predict 30-day mortality rates in hospitalized CAP patients, better than those of CURB-65 and PSI. Our study revealed that combining fetuin-A level with CURB-65 and PSI significantly improved the accuracy of SCAP prediction. Combining fetuin-A level with CURB-65 and PSI significantly improved the accuracy of SCAP prediction. These data demonstrate that fetuin-A is a valuable biomarker that may be used to identify patients at high risk of SCAP development. Fetuin-A was significantly and negatively associated with CRP level, which is consistent with a previous study that assessed fetuin-A effects of inflammation (26, 32). It is an effective indicator of CAP prognosis.

Throughout the human lifespan, fetuin-A levels tend to be highest at infancy, and slightly decrease throughout childhood and adulthood, after which point they remain relatively constant (33). These differences may lead affect findings when patients of different age groups are assessed, and the specific mechanism is not fully understood.

Fetuin-A is regulated by different proinflammatory mediators. The protein functions in the acute-phase response to injury and infection, and has emerged as a multifaceted protective factor that locally counteracts calcification, modulates macrophage polarization, and attenuates inflammation and fibrosis (34–36). High mobility group box-1 (HMGB1) induces innate immune cellular expression of various cytokines, chemokines, and adhesion molecules to sustain a potentially injurious level of systemic inflammation. Fetuin-A directly inhibits HMGB1 release (34, 37). Further, fetuin-A plays a protective role against oxidative stress (38). However, the exact mechanism by which fetuin-A levels are decreased in patients with CAP remains unknown. Given the multiple roles of fetuin-A, the precise pathway by which fetuin-A functions in CAP and its potential relevance as a bioindicator deserves further exploration.

This study has two main limitations. First, healthy individuals served as the control group. The influence of other diseases on plasma fetuin-A levels could not be ruled out, and evidence for a diagnosis of CAP was slightly insufficient. Therefore, this study mainly focused on the prognostic and severity grading value of fetuin-A in CAP. Second, plasma fetuin-A levels were detected at the time of admission and only a small part of discharge, and a lack of complete paired data.

In conclusion, our results demonstrated that plasma fetuin-A levels were reduced in patients with CAP vs. healthy controls, and particularly in non-survivors and SCAP. Fetuin-A level could be used to accurately predict 30-day mortality in patients with CAP. Incorporation of fetuin-A improves the prognostic accuracy of CURB-65 and PSI. Early and ongoing detection of plasma fetuin-A levels in patients with CAP provides prognostic information, and has the potential facilitate the identification of high-risk patients.

## Data availability statement

The original contributions presented in the study are included in the article/supplementary material, further inquiries can be directed to the corresponding authors.

## Ethics statement

The studies involving human participants were reviewed and approved by Medical Ethics Committee of Peking University People's Hospital (approval no.: 2016PHB202-01). The patients/participants provided their written informed consent to participate in this study.

## Author contributions

LZ, YS, and ZG designed the study. LZ performed ELISA experiment. XM, WN, YH, and DY acquired clinical data. LZ, YS, QL, and XM performed data analysis. LZ and YS drafted the manuscript. YX and ZG obtained research funding. All authors read and approved the final manuscript.

## Funding

This work was supported by grants from the Chinese Science and Technology Key Project (2017ZX10103004-006), the National Key Research and Development Programme of China (2016YFC0903800), and the National and Provincial Key Clinical Specialty Capacity Building Project 2020 (Department of the Respiratory Medicine).

## Conflict of interest

The authors declare that the research was conducted in the absence of any commercial or financial relationships that could be construed as a potential conflict of interest.

## Publisher's note

All claims expressed in this article are solely those of the authors and do not necessarily represent those of their affiliated organizations, or those of the publisher, the editors and the reviewers. Any product that may be evaluated in this article, or claim that may be made by its manufacturer, is not guaranteed or endorsed by the publisher.
