# Supplementary material for: Decreased plasma fetuin-A level as a novel bioindicator of poor prognosis in community-acquired pneumonia: A multi-center cohort study
Source: Front Med (Lausanne). 2022 Jul 29;9:807536. doi: 10.3389/fmed.2022.807536 (PMC9372348; doi:10.3389/fmed.2022.807536)
Supplement: Supplementary file 1 [file Data_Sheet_1.docx]

**Supplementary Table 1. AUC and thresholds for predicting non-survivors with survivors**

|  | Threshold | Sensitivity (%) | Specificity (%) | AUC | P value | 95% CI  Lower limit | Higher limit |
| --- | --- | --- | --- | --- | --- | --- | --- |
| WBC | >13.6×10^3^/mm^3^ | 42.11 | 91.25 | 0.588 | 0.3244 | 0.528 | 0.646 |
| NE% | >83.2% | 84.21 | 76.05 | 0.793 | <0.0001 | 0.741 | 0.839 |
| Albumin (ALB) | ≤30 g/L | 68.42 | 78.91 | 0.761 | 0.0001 | 0.706 | 0.810 |
| CRP | >77.9 mg/L | 84.21 | 67.37 | 0.746 | <0.0001 | 0.687 | 0.798 |
| PCT | >0.12 µg/L | 94.74 | 52.77 | 0.727 | <0.0001 | 0.668 | 0.781 |
| PSI | >76 | 94.74 | 48.48 | 0.745 | <0.0001 | 0.690 | 0.794 |
| CURB-65 | >1 | 57.89 | 83.33 | 0.754 | <0.0001 | 0.699 | 0.803 |
| Fetuin-A | ≤309 mg/L | 94.74 | 68.94 | 0.871 | <0.0001 | 0.826 | 0.907 |
| AUC, area under the curve; CI, confidence interval; WBC, White blood cell; NE%, percentage of neutrophils; CRP, C-reactive protein; PCT, procalcitonin; PSI, Pneumonia Severity Index; CURB-65, confusion, urea > 7 mmol/L, respiratory rate ≥ 30 breaths/min, low blood pressure and age ≥ 65 years. | | | | | | | |

**Supplementary Table 2.** Clinical characteristics and laboratory findings of SCAP and Non-SCAP

|  | Non-SCAP  n=226 (79.86%) | SCAP  n=57 (20.14%) | P value |
| --- | --- | --- | --- |
| Male sex (%) | 131 (57.96%) | 43 (75.44%) | 0.015 |
| Age (years) | 66 (54-77) | 68(54-77) | 0.618 |
| Comorbidities, n (%) |  |  |  |
| Heart disfunction | 20 (8.85%) | 3 (5.26 %) | 0.539 |
| Chronic renal disease | 10 (4.42%) | 5 (8.77%) | 0.328 |
| Liver disease | 10(4.42%) | 2 (3.51%) | 1.000 |
| Diabetes mellitus | 51 (22.57%) | 10 (17.54%) | 0.339 |
| High pressure | 76 (33.63 %) | 20 (35.09 %) | 0.835 |
| Laboratory findings |  |  |  |
| WBC count (×10^3^/mm^3^) | 6.60 (4.93-9.76) | 11.31(5.39-17.95) | 0.0001 |
| NE% | 80.05 (70.10-86.92) | 88.40 (81.80-92.00) | <0.0001 |
| Hemoglobin level (g/dL) | 129.00 (117.00-140.00) | 127.00 (111.50-141.00) | 0.669 |
| Platelet count (×10^3^/mm^3^) | 207.00 (148.00-276.00) | 186.00 (124.50-276.50) | 0.241 |
| Glucose (mmol/L) | 5.42 (4.81-5.87) | 6.80 (5.30-11.04) | 0.0002 |
| Albumin (g/L) | 36.00 (32.98-40.00) | 29.00 (25.50-33.50) | <0.0001 |
| Blood urea nitrogen (mmol/L) | 4.60 (3.61-6.40) | 5.90 (5.00-10.70) | <0.0001 |
| CRP (mg/L) | 35.00 (7.39-86.70) | 136.50 (81.63-140.50) | <0.0001 |
| PCT (µg/L) | 0.08 (0.05-0.38) | 1.78 (0.41-6.21) | <0.0001 |
| Complications, n (%) |  |  |  |
| Sepsis | 3 (1.33%) | 31 (54.39%) | <0.0001 |
| Pleural effusion | 66 (29.20%) | 36 (63.16) | <0.0001 |
| ARDS | 2 (0.88%) | 14(24.56%) | <0.0001 |
| Confusion | 3 (1.35%) | 9 (16.36%) | <0.0001 |
| Non-invasive ventilation, n (%) | 124(54.87%) | 24(42.11%) | 0.539 |
| Invasive ventilation, n (%) | 0(0.00%) | 32(100.00%) | <0.0001 |
| ICU admission, n (%) | 7(3.10%) | 44(77.19%) | <0.0001 |
| Pathogens n (%) |  |  |  |
| Bacteria | 60 (22.73%) | 3 (15.79%) | 0.677 |
| Virus | 75 (28.41%) | 4 (21.05%) | 0.490 |
| Fungus | 19(7.20%) | 1 (5.26%) | 1.000 |
| Mixed | 82(31.06%) | 8(42.11%) | 0.318 |
| Drug treatment, n (%) |  |  |  |
| Antibiotics | 221(97.79%) | 57(100.00%) | 0.568 |
| Antiviral drugs | 18(7.96%) | 26(45.61%) | <0.0001 |
| Corticosteroids | 26(14.39%) | 21(47.37%) | <0.0001 |
| PSI | 74 (55-90) | 108 (86-128) | <0.0001 |
| CURB-65 | 1 (0-1) | 2 (1-2) | <0.0001 |
| non-survivors, n (%) | 1 (1.44%) | 18 (31.58%) | <0.0001 |
| Data are presented as means ± standard deviation from the mean, or median (interquartile range) or n (%). WBC, White blood cell; NE%, percentage of neutrophils; CRP, C-reactive protein; PCT, procalcitonin; ARDS, Acute Respiratory Distress Syndrome; PSI, Pneumonia Severity Index; CURB-65 confusion, urea >7 mmol/L, respiratory rate ≥30 breaths/min, low blood pressure, and age ≥65 years. | | | |

**Supplementary Table 3.** Logistic regression analysis of risk factors associated with SCAP

|  | Univariate analysis |  | Multivariate analysis^#^ |  |
| --- | --- | --- | --- | --- |
|  | Odd ratio (95% CI) | P value | Odd ratio (95% CI) | P value |
| PSI |  |  |  |  |
| PSI (Ⅰ-Ⅲ) | reference |  |  |  |
| PSI (Ⅳ) | 5.464 (2.759-10.819) | <0.0001 | 5.829 (1.775-19.149) | 0.004 |
| PSI (Ⅴ) | 20.944 (7.097-61.813) | <0.0001 | 2.009 (0.344-11.749) | 0.439 |
| CURB-65 |  |  |  |  |
| CURB-65 (0-1) | reference |  |  |  |
| CURB-65 (2-5) | 11.297 (5.735-22.255) | <0.0001 | 26.393 (7.235-96.289) | <0.0001 |
| Fetuin-A |  |  |  |  |
| Fetuin-A (≥455.64) | reference |  |  |  |
| Fetuin-A (≥365.92, <455.64) | 1.333 (0.287-6.184) | 0.713 | 0.799 (0.096-6.668) | 0.836 |
| Fetuin-A (≥271.54, <365.92) | 4.690 (1.262-17.429) | 0.021 | 3.948 (0.685-22.770) | 0.124 |
| Fetuin-A (≥202.86, <271.54) | 17.000 (4.597-62.869) | <0.0001 | 13.761 (2.303-82.229) | 0.004 |
| Fetuin-A (<202.86) | 56.667 (13.732-233.845) | <0.0001 | 17.864 (2.651-120.394) | 0.003 |
| CI, Confidence interval; WBC, White blood cell; NE%, percentage of neutrophils; CRP, C-reactive protein; PCT, procalcitonin; PSI, Pneumonia Severity Index; CURB-65, confusion, urea >7 mmol/L, respiratory rate ≥30 breaths/min, low blood pressure and age ≥65 years. ^#^Adjusted for Male sex, Age, WBC, NE%, Hemoglobin level, Platelet count, Glucose, Albumin, Blood urea nitrogen, CRP, PCT. | | | | |

**Supplementary Table 4. AUC and thresholds for predicting SCAP with Non-SCAP**

|  | Threshold | Sensitivity (%) | Specificity (%) | AUC | P value | 95% CI  Lower limit | Higher limit |
| --- | --- | --- | --- | --- | --- | --- | --- |
| WBC | >10.63×10^3^/mm^3^ | 56.14 | 82.22 | 0.664 | 0.0010 | 0.605 | 0.719 |
| NE% | >81.6% | 78.95 | 79.56 | 0.851 | <0.0001 | 0.804 | 0.890 |
| Albumin (ALB) | ≤32 g/L | 73.68 | 78.44 | 0.782 | <0.0001 | 0.728 | 0.829 |
| CRP | >91.7 mg/L | 73.08 | 77.83 | 0.788 | <0.0001 | 0.733 | 0.836 |
| PCT | >0.31µg/L | 81.82 | 71.86 | 0.822 | <0.0001 | 0.769 | 0.867 |
| PSI | >80 | 87.72 | 62.39 | 0.819 | <0.0001 | 0.769 | 0.862 |
| CURB-65 | >1 | 56.14 | 89.82 | 0.800 | <0.0001 | 0.749 | 0.845 |
| Fetuin-A | ≤279 mg/L | 73.68 | 84.51 | 0.836 | <0.0001 | 0.787 | 0.877 |
| CURB-65 _ Fetuin-A |  | 80.70 | 85.84 | 0.901 | <0.0001 | 0.861 | 0.934 |
| PSI _ Fetuin-A |  | 85.96 | 77.43 | 0.889 | <0.0001 | 0.847 | 0.923 |
| AUC, area under the curve; CI, confidence interval; WBC, White blood cell; NE%, percentage of neutrophils; CRP, C-reactive protein; PCT, procalcitonin; PSI, Pneumonia Severity Index; CURB-65, confusion, urea > 7 mmol/L, respiratory rate ≥ 30 breaths/min, low blood pressure and age ≥ 65 years. | | | | | | | |

**Supplementary Table 5. AUC and thresholds for predicting non-survivors with survivors**

|  | Threshold | Sensitivity (%) | Specificity (%) | AUC | P value | 95% CI  Lower limit | Higher limit |
| --- | --- | --- | --- | --- | --- | --- | --- |
| Fetuin-A _ admission | <296.65 mg/L | 100.00 | 78.26 | 0.899 | <0.0001 | 0.783 | 0.965 |
| Fetuin-A _ discharge | <271.39 mg/L | 100.00 | 93.48 | 0.989 | <0.0001 | 0.911 | 1.000 |
| Fetuin-A _ change | <-40.64 mg/L | 83.33 | 80.43 | 0.808 | <0.0001 | 0.675 | 0.904 |
| Fetuin-A _ discharge and change |  | 100.00 | 97.83 | 0.993 | <0.0001 | 0.932 | 1.000 |
| AUC, area under the curve; CI, confidence interval; Fetuin-A _ admission, Fetuin-A level at admission l; Fetuin-A _ discharge, Fetuin-A level at discharge; Fetuin-A _ change, Fetuin-A level at discharge minus Fetuin-A level at admission; Fetuin-A _ discharge and change, Fetuin-A level at discharge combined with Fetuin-A _ change. | | | | | | | |
